# Immunohistochemical localization of hepatopancreatic phospholipase in gastropods mollusc, *Littorina littorea *and *Buccinum undatum *digestive cells

**DOI:** 10.1186/1476-511X-10-219

**Published:** 2011-11-25

**Authors:** Zied Zarai, Nicholas Boulais, Pascale Marcorelles, Eric Gobin, Sofiane Bezzine, Hafedh Mejdoub, Youssef Gargouri

**Affiliations:** 1 Laboratoire de biochimie et de génie enzymatique des lipases, ENIS BPW 1173 Université de Sfax-Tunisia; 2 Laboratoire de Neurobiologie Cutanée, CHU Morvan, Université de Brest, 29609 BREST cedex France; 3 Service d'Anatomie Pathologique, Pôle de Biologie-Pathologie, CHU Morvan, Université de Brest, 29609 BREST cedex France

**Keywords:** phospholipase A_2_, digestive enzyme, *littorina littorea*, *Buccinum undatum *hepatopancreas, immunolocalisation

## Abstract

**Background:**

Among the digestive enzymes, phospholipase A_2 _(PLA_2_) hydrolyzes the essential dietary phospholipids in marine fish and shellfish. However, we know little about the organs that produce PLA_2_, and the ontogeny of the PLA_2_-cells. Accordingly, accurate localization of PLA_2 _in marine snails might afford a better understanding permitting the control of the quality and composition of diets and the mode of digestion of lipid food.

**Results:**

We have previously producted an antiserum reacting specifically with mSDPLA_2_. It labeled zymogen granules of the hepatopancreatic acinar cells and the secretory materials of certain epithelial cells in the depths of epithelial crypts in the hepatopancreas of snail. To confirm this localization a laser capture microdissection was performed targeting stained cells of hepatopancreas tissue sections. A Western blot analysis revealed a strong signal at the expected size (30 kDa), probably corresponding to the PLA_2_.

**Conclusions:**

The present results support the presence of two hepatopancreatic intracellular and extracellular PLA_2 _in the prosobranchs gastropods molluscs, *Littorina littorea *and *Buccinum undatum *and bring insights on their localizations.

## Background

Snails require lipids for metabolic energy and for maintaining the structure and integrity of cell membranes in common with other animals to tolerate environemental strains [1]. The analyses of lipid composition of digestive gland and pedal muscle of two northern freshwater pulmonate snails *Lymnaea stagnalis *and *Lymnaea ovata *and three marine prosobranch gastropods *Littorina obtusata, Littorina littorea *and *Buccinum undatum *from the White Sea, shown that the content of triacylglycerides both in digestive gland and pedal was higher in littoral dwellers *Littorina*, the activity of which depends on the tide level. The presence of massive shell enhances demands in energy needed for supporting movement and activity. Because the intensity of energy metabolism is related to quantity of total phospholipids, mitochondria and activity of their oxidizing ferments, the presence of thick shell in marine snails together with motor activity costs more in terms of energy than in freshwater snails with thin shell [1].

In different molluscs, food is processed to varying degrees as it passes through the alimentary tract. It is generally assumed that digestion of ingested material takes place in two phases, an extracellular process and intracellular digestion, where the prevalence of one over the other depends on the type of diet of the animal.

In general terms, the digestive glands of most molluscs present a common organization and a single epithelium comprised by at least two cell types, namely, digestive and basophilic cells found in the digestive diverticula [2]. Digestive cells are involved in the intracellular digestion of food and possess a highly developed endo-lysosomal system, whereas basophilic cells are secretory cells with a highly developed rough endoplasmic reticulum [3].

Although the digestive enzymes are well characterized, including pepsin, trypsin, chymotrypsin, and amylase, little information is available on the lipid digestive enzymes: lipases and phospholipases. This is mostly due to difficulties in purification and histochemical analysis of the enzymes in fish [4].

Among the lipid digestive enzymes, phospholipases A_2 _(PLA_2_; EC3.1.1.4) is potentially important in marine snails, for hydrolysis of the essential dietary phospholipids. PLA_2 _catalyzes selective hydrolysis of the sn-2 acyl ester bond in 1,2-diacyl-sn-glycero-3 phospholipids, resulting in the formation of lysophospholipids and free fatty acids [5]. The occurrence, properties and physiological role of various PLA_2 _in aquatic organisms have been explained in several publications. Non-specific lipid acylhydrolases exhibiting combined action of various lipases such as phospholipases have also been recovered and examined from aquatic organisms [6-8].

This study describes immunohistochemically analysis of PLA_2 _in the hepatopancreas organs of the adults' marine snail *littorina littorea *and *Buccinum undatum *using an antiserum against *Hexaplex trunculus *hepatopancreatic PLA_2 _[9].

## Results

### Morphological analysis of digestive epithelium

**Figure 1:**
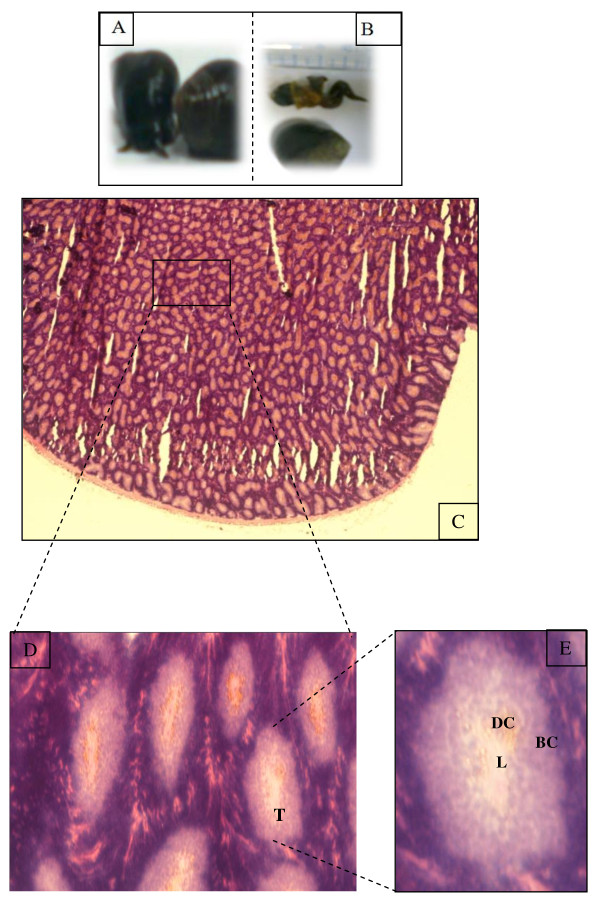
***Littorea. littorina***. (A), *Littorea. littorina fished in Franch Brittany coast*. (B), the shell has been removed in the posterieur side taking care to keep the mantle is intact. The marine snail sagittal section exhibited the hepatopancreas in the posterieur side. (C), light microscopic view of sections of digestive glands. Sections were stained with hematoxylin-eosin for observing the general morphology. (D and E), magnification of the digestive diverticula sectioned longitudinally. T: digestive tubule. DC: digestive cell. BC: basophilic cell. L: lumen of digestive tubule.

The digestive gland of the gastropod snail, *Littorina littorea *consists of blind ending tubules composed of basophilic and digestive cells (Figure 1) [10,11]. The function of the digestive cells is the endocytosis and the intracellular digestion of food material, conveyed to them from the stomach via the tubule lumina, and for this purpose they have a well developed lysosomal-vacuolar system [12,13].

*Buccinum undatum*, the northern whelk, is a common snail of moderate size (8 cm) on the northeastern coast of North America and in northern Europe. Several other species occur in the Pacific Northwest. *Buccinum undatum *is commercially harvested for human consumption in Europe.

**Figure 2:**
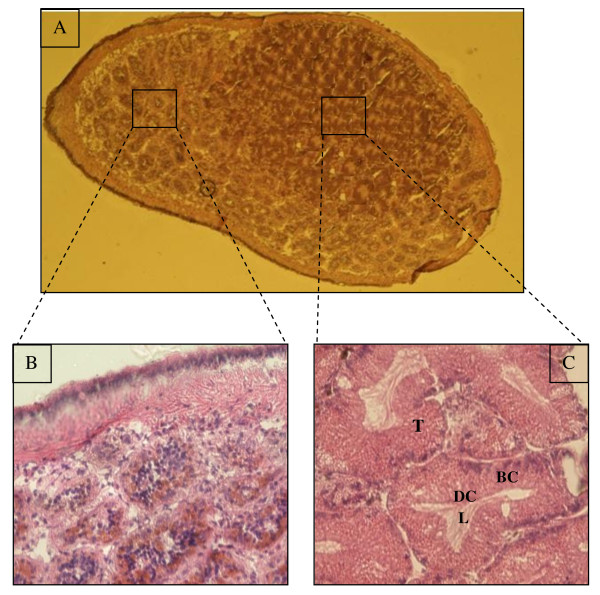
***Buccinum undatum***. (A), Transversal section of *Buccinum undatum*. (B and C) (Tétreault et al., 2000), the shell has been removed in the posterieur side but the mantle is intact, marine snail sagittal section showing the hepatopancreas in the posterieur side. (A), *Buccinum undatum *hepatopancreas showing the epitleluim of the digestive diverticula which displayed a small lumen through and the interstitial tissue. Sections were stained with hematoxyline-eosine. (B and C), Enlarged view of the digestive diverticula sectioned longitudinally. T: digestive tubule. DC: digestive cell. BC: basophilic cell. L: lumen of the digestive tubule.

The digestive gland (Figure 2) is a vast pocket related to stomach by the only one ciliate opening, it lacks well differentiated channels collectors. In the neighborhood of stomach, it is partially divided up by folds of it wall. In the posterior zone towards the apex of the twist, the partitions join to bound tubules. Every tubule of 30 μm approximately, rest on a fine basal blade. Light tubules are wide and cavities represent an important fraction of the volume of organ and composed of basophilic and digestive cells.

### Specificity of antiserum to *Hexaplex trunculus *PLA_2_

**Figure 3:**
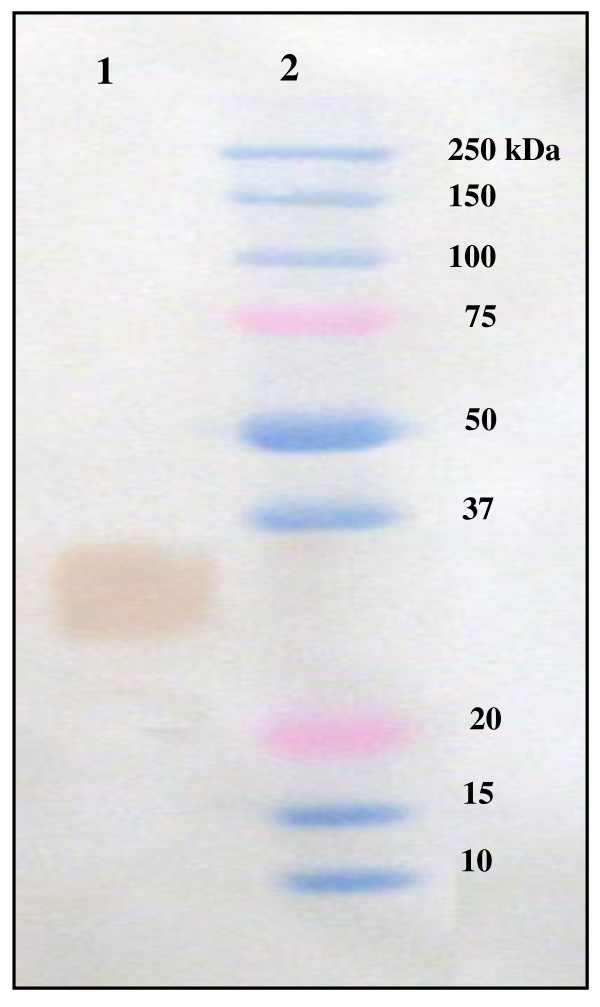
**Western blot analysis**. Western blot analysis of 20 μg hepatopancreatic snail extract (lane 1). The revelation was carried out using mSDPLA2 antiserum at 1: 1000. Lane 2: Proteins markers.

The supernatant of the marine snail hepatopancreas homogenate containing 100 μg of total proteins was subjected to SDS-PAGE analysis followed by immunoblotting. The anti-mSDPLA_2 _polyclonal antibody was found to react with a single band of 30 kDa corresponding to mSDPLA_2 _present in the crude extract (Figure 3). No other proteins react with anti-mSDPLA_2 _sera, suggesting a good specificity of our mSDPLA_2 _antiserum. Based on its specificity towards mSDPLA_2_, the polyclonal antibodies were used for immunocytolocalization of PLA_2 _in the hepatopancreas tissue of the prosobranchs gastropods molluscs, *Littorina littorea *and *Buccinum undatum*.

### Histological, immunochemical and immunofluorescence studies

The location of the digestive PLA_2 _in marine snail *Littorina littorea *and *Buccinum undatum *was studied immunohistochemically by using an antiserum against the *Hexaplex trunculus *digestive mSDPLA_2_. The antiserum efficiently react with hepatopancreatic cells of marine snail *Littorina littorea *and *Buccinum undatum*.

**Figure 4:**
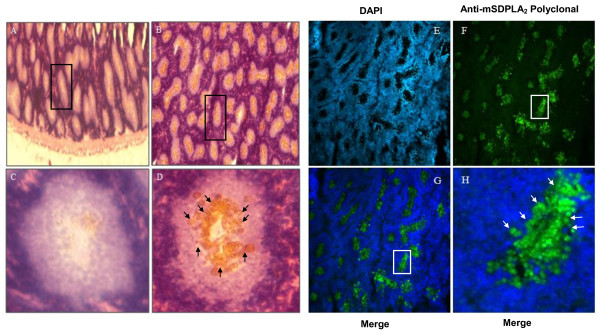
**Section of digestive gland showing mSDPLA2 immunoreactivity in digestive tubules and immunofluorescence localization of phospholipase in a 4-μm frozen section**. (A and C) control satining in absence of primary antiserum. (B, D) Few mSDPLA2 positive nuclei were detected. These immunoreactive nuclei correspond mainly to digestive cells (arrows). Basophilic cells remained unstained (D). (E) One section was first stained with DAPI for general morphology. (F) pAbs anti mSDPL (1:100) revealed by fluorochrome (chromeo™488) conjugated to secondary antibody diluted 1:200. (G and H) merge pictures of (E and F), showing labeling of intracellular granules of digestive cells. Sections of digestive glands showed immunoreactivity to mSDPLA2 immunoreactivity in digestive tubules. Few mSDPLA2 positive nuclei appeared.

**Figure 5:**
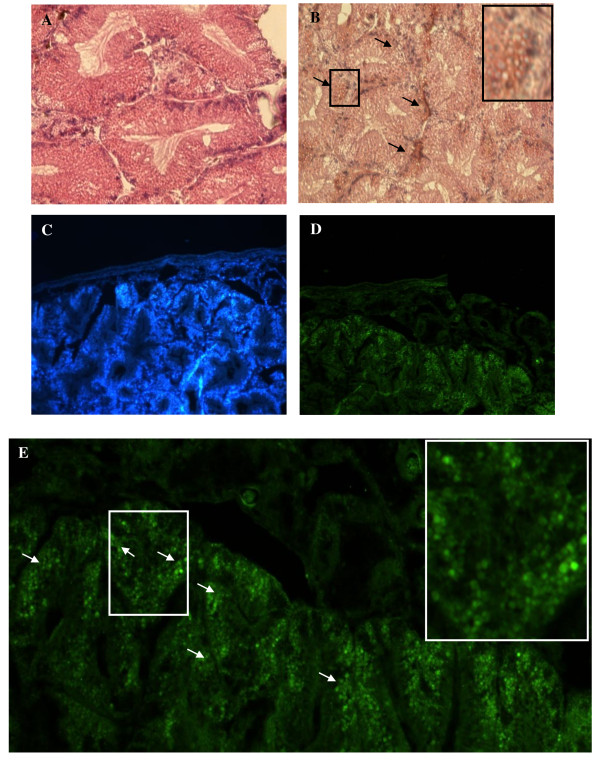
**Immunohistochemistry and immunofluorescence localization of phospholipase in a 4-μm frozen sections**. (A) Sections of digestive glands showing mSDPLA2 immunoreactivity in digestive tubules. Few mSDPLA2 positive nuclei appeared. These immunoreactive nuclei corresponded mainly to secretory cells (arrows), whereas the nucleus of digestive cells was mSDPLA2 negative (B). (B) Control section of digestive gland incubated in absence of anti- mSDPLA2 antiserum. Any reaction was disclosed. (C) One section was first counter-stained with DAPI for observing general morphology. (D and E) followed by pAbs anti-mSDPL antiserum (1:100) revealed by fluorochrome (chromeo™488) conjugated to the secondary antibody diluted 1:200 in PBS.

Only digestive cells of *Littorina littorea *displayed a positive labeling for the presence of mSDPLA_2_. Conversely, secretory zymogene-like cells were not immunostained (Figure 4). Interestingly, we noticed that only few intracellular granules on the digestive cells were immunoreactive to anti-mSDPLA_2_. These granules with irregular in shape did not have a specific location in the digestive diverticula; they were tentatively named phospholipase granules. However, the basophilic cells in the hepatopancreas of *Buccinum undatum *were immunoreactive to the *Hexaplex trunculus *PLA_2 _antibodies. No labeling was detected in the digestive cells (Figure 5). The granular labeling observed in the basophilic cells indicates that the PLA_2 _might be an extratracellular enzyme involved in the extratracellular food digestion process.

**Figure 6:**
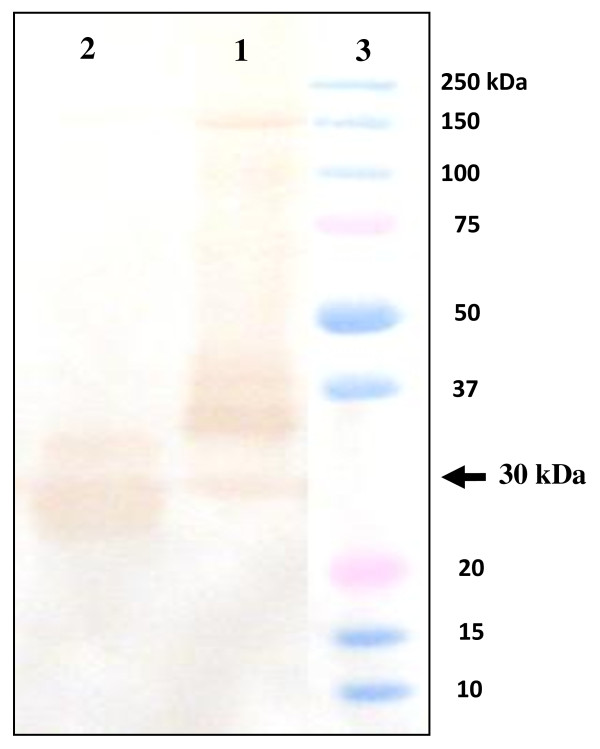
**A Laser captures microdissection**. A Laser captures microdissection targeting stained cells of hepatopancreas tissue sections was performed. Selected cells were dissociated and protein extracts were analyzed by western-blot. pAbs anti-mSDPLA_2 _reacted with the homogenate of stained cells of hepatopancreatic tissue sections of *Buccinum undatum *(lane 1) and *Littorina littorea *(lane 2). Lane 3: Proteins markers.

To confirm the presence of PLA_2 _and the specificity of the mSDPLA_2 _polyclonal antibodies, we performed a laser capture microdissection targeting stained cells of hepatopancreas tissue sections. Selected cells were dissociated and proteins were extracted for immunobloting analysis. Western blotting revealed a strong broad band at around 30 kDa for both snails *Littorina littorea *and *Buccinum undatum*, corresponding probably to the PLA_2 _(Figure 6).

## Discussion

### Phospholipase A2 localization in the hepatopancreas snails

The digestive diverticulum consists of an epithelium with a single layer of cells, separated from the surrounding connective tissue and muscle cells by a basal lamina. In several molluscs these epitheliums correspond to the digestive and basophilic cells [14-17]. However, in gastropods more cell types have been reported [18-20].

The digestive gland is composed of two main cell types, the "digestive" cells and the "secretory" cells. Digestive cells appear to be involved in the absorption and digestion of nutrients, while secretory cells produce digestive enzymes and calcareous concretions. Undifferentiated cells are scattered between these two cell types.

In the present work, we reported the presence of hepatopancreatic PLA_2 _in the digestive gland of prosobranch gastropod mollusc, *Littorina littorea *and *Buccinum undatum*. We have performed immunocytochemical and immunofluorescence analysis to specifiy the tissular and subcellular location of this digestive enzyme. We showed that in the hepatopancreas of the *Littorea littorina*, digestive cells were immunoreactive to the *Hexaplex trunculus *PLA_2 _antibody whereas no labeling was detected in the basophilic cells. From the granular labeling observed in digestive cells, we suggested that PLA_2 _might be an intracellular enzyme involved in the intracellular food digestion process as described for other invertebrates [21]. In mammals, classical digestive enzymes as pancreatic enzymes were detected in intracellular zymogene granules in pancreatic acinar cells. However they act in the lumen of the gastrointestinal tract which requires a secretory process [22]. The presence of mSDPLA_2 _revealed in digestive cells is not a definitive argument for phospholipid digestion mechanism located inside these cells. Invertebrate digestive cells can uptake partially digested food by endocytosis through microvilli. It was demonstrated that digestive cells of a mollusc *Sepia officinalis *absorb 80% of a radiolabeled food source [23]. According to Boucaud-Camou and Yim [24], the pinocytotic vesicles fuse together to form heterogeneous phagosomes known as heterophagosomes. When they combine with primary lysosomes containing the intracellular digestive enzymes, they form secondary lysosomes or a heterolysosomes, where in the intracellular digestion takes place. We noted that phospholipase granules belonging to the digestive cells were irregular in shape and size.

In the hepatopancreas of *Buccinum undatum*, basophilic cells were immunoreactive to the mSDPLA_2 _antibodies (Figure 5); while no labeling was observed in the digestive cells. From the granular labeling observed in the basophilic cells, we suggested that PLA_2 _might be an extratracellular enzyme involved in the extratracellular food digestion process as described for other invertebrates [25].

The basophilic cells seem to be responsible for the digestive enzymes secretions that undertake an initial and rapid extracellular food hydrolysis in the diverticula lumen. Then, the partial digestion products are likely absorbed by pinocytosis and stored in the digestive cell until they are slowly hydrolyzed by intracellular enzymes for energy generation.

Yonge (1926) [25] considered the relative importance of extra- and intracellular digestion in the oyster, *Ostrea edulis *and showed that diet digestion was intracellular, in amoebocytes or in digestive gland cells. Yonge (1926) felt that the only extracellular enzymes of consequence were carbohydrases from the crystalline style, and that the traces of proteases and lipases found in the stomach were derived from burst phagocytes. Since that time, there has been a growing appreciation of the importance of extracellular digestive processes in bivalves. Moreover, George (1952) [26] showed that extracellular lipolysis occurs in the stomachs of *Crassosirea virginica and Geukensia demissa*. Mansour *(*1946) [27] found significant amounts of proteolytic and lipolytic enzymes in lamellibranch stomachs. These enzymes originated in spheres periodically pinched off and excreted from digestive gland cells.

### Electron microscopic study of digestive cells

**Figure 7:**
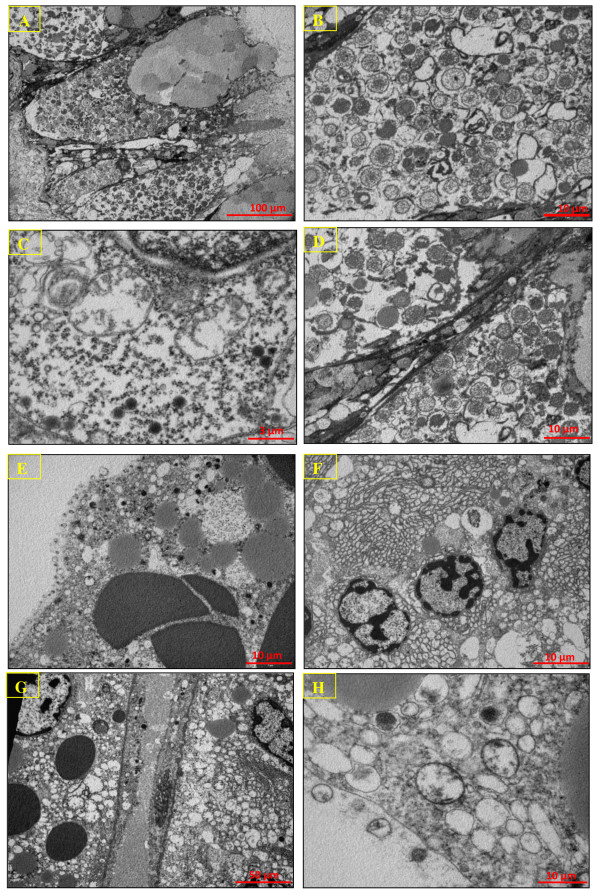
**Transmission Electron Microscopy**. Transmission Electron Microscopy of the digestive cells of *Littorina littirea *(A, B, C and D) and basophilic cells of *Buccinum undatum *(E, F, G and H).

The ultrastructural morphology of digestive cells in *Littorina littirea *has been previously described [13]. Briefly, the cells are columnar with an apical microvillous luminal border and basal nucleus (Figure 7A). They are dominated by large macrovisicles of a major compartement of the lysosomal-vacuolar system, needed for intracellular digestion of food absorption, and storage of lipids.

Digestive cells have externely large heterolysosomes (Figure 7A,B,C and 7D). Residual bodies occurred predominantly in the basal area of the cells and often showed evidence of transfer of contents from the heterolysosomes (Figure 7A). Lipid inclusions and lipofuscin granules were also located typically in the basal region of the cells (Figure 7B). Mithochondria and occasional Golgi bodies occurred along the periphery of the cells in close association with the heterolysosomes. There were evidences for either the presence of canals for linking pinocytotic vesicles to heterophagosomes or alternatively the formation of 'transport vesicles' from the heterophagosomes to heterolysosomes (Figure 7C and 7D) as discussed previously [13,16].

In semi-thin sections of *Buccinum undatum *hepatopancreas, many secretory vesicles were observed in the digestive diverticula epithelium. Basophilic cells exhibit numerous heterolysosomes sometime fused together or with phagosomes (Figure 7E and 7F). The apical surface of basophilic cells was covered with microvilli, almost reaching 2 μm in length (Figure 7E). We describe in this work a second cell types for which a single large vacuole occupying almost entirely the cell, while the cytoplasm is reduced to a very thin peripheral layer (Figure 7F).

Many endocytic vesicles filled with electron dense materials were observed in the apical region of *Buccinum undatum *basophilic cells. Probably, these vesicles contained extracellular digestion products that would be transferred to heterolysosomes of digestive cells, to complete the digestive process. In some basophilic cells the number of endocytic vesicles was very high, indicating a very intense endocytic activity (Figure 7H). Conversely, some other cells had, only few vesicles. Suggesting different digestive steps at the cellular level, with low endocytic activity. The Golgi stacks with dilated cisternae contained dense substances detected in basophilic cells of *Buccinum undatum *(Figure 7G).

Small and electron dense vesicles were associated with secretory functions, whereas large and electron-lucid vesicles were associated with absorption functions. Furthermore, the presence of lipid droplets and glycogen granules in the cytoplasm of basophilic cells suggests the involvement of these cells in the lipids metabolism (Figure 7G).

The thorough involvement of the hepatopancreas of *Buccinum undatum *in secretion, digestion, adsorption and metabolism was evidenced by the decondensed aspect of nuclear chromatin so reflecting an intense transcriptionnal activity, confirmed by the presence of rough endoplasmic reticulum, Golgi complex region, lysosomes, vesicles and cytoplasmic inclusions in the basophilic cells. The cytoplasm of basophilic cells contained granules, vesicles with protein content and lipid droplets, indicating possible function in secretion and highlight that extracellular lipolysis should occur in basophilic cells of *Buccinum undatum*. The finding of significant amounts of lipolytic enzymes in lamellibranch hepatopancreas suggest that these enzymes originated in spheres periodically pinched off and excreted from digestive glandular cells.

### Laser capture microdissection and western blot analysis

The purified *Hexaplex trunculus *mSDPLA_2 _and homogenate PLA_2 _from the marine snails (*littorina littorea *and *Buccinum undatum*) hepatopancreas were run electrophoretically on SDS PAGE without reducing agent. A signal of 30 kDa in weight was detected in each specie by NBT-BCIP method (Figure 6). The antiserum against *Hexaplex trunculus *mSDPLA_2 _immunoreacts with the hepatopancreatic PLA_2 _of prosobranch gastropod mollusc, *Littorina littorea *and *Buccinum undatum*.

As expected, signal of about 30 kDa was obtained, pAbs anti-mSDPLA_2 _was found to react specifically with a 30 kDa protein band corresponding probably to the phospholipase. This molecular mass is comparable to that of hepatopancreatic digestive mSDPLA_2 _of marine snail *Hexaplex trunculus*[9]. Since the mSDPLA_2 _antiserum was prepared from the marine snail *Hexaplex trunculus*, the small deffense in size obtained here is likely due to variation in length and sequence between species. In addition, the antiserum obviously produced a low background and some unspecific signals but at lower degree compared to the 30 kDa bands.

## Materials and methods

### Animals collection

Marine snails' *littorina littorea *and *Buccinum undatum *were collected from the Atlantic coasts of French Brittany. They were kept on ice until use. Only the hepatopancreases were collected immediately and stored at -80°C.

### Preparation of the antiserum to *Hexaplex trunculus *PLA_2_

Antibodies against mSDPLA_2 _were produced in rabbits by three biweekly subcutaneous injections of 250 μg of purified mSDPLA_2 _emulsified with complete Freund's adjuvant. Sera were obtained from blood samples two weeks after the last injection. Reactivity of collected antiserum was determined by the microplate ELISA method using the *Hexaplex trunculus *mSDPLA_2_.

### SDS-PAGE and immunoblotting technique

Analytical polyacrylamide gel electrophoresis of proteins in the presence of sodium dodecyl sulfate (SDS-PAGE) was performed according to the Laemmli's method [28]. The specificity of pAbs anti-mSDPLA_2 _was established by protein blotting. Proteins from SDS gel were transferred to nitrocellulose membranes. After the transfer, membranes were rinsed three times in PBS (10 mM phosphate, NaCl 150 mM, pH 7.2), with 3% half-fat milk for 1 h at room temperature. Thereafter, membranes were incubated with pAbs anti-mSDPLA_2 _diluted at 1:1000 in PBS containing 0.05% tween-20 (PBS-T) for 1 h at room temperature. Afterward, they were washed three times with PBS-T and incubated for 1 h at room temperature with a 1:2000 dilution of alkaline phosphatase-conjugated anti-rabbit immunoglobulin (Sigma). Then, washing as above, the revelation were carried out using a NBT-BCIP kit according to the supplier (Sigma).

### Histology, immunochemistry and immunofluorescence

#### Immunohistochemistry

Tissues, with a size of around 1 mm^3^, were embedded in Optimal Cutting temperature (OCT) and cryopreserved in isopentane chilled on liquid nitrogen. Sections with a thickness of 4 μm were cut. Slides were saturated with 5% of normal goat serum in PBS with 0.05% Triton X-100 for 15 minutes and subsequently hybridized with primary anti-mSDPLA_2 _polyclonal antibodies diluted at 1:200 in Dako Diluent (S3022) for 2 h at 4°C. Slides were rinsed twice and hybridized with secondary anti-rabbit biotinylated- antibodies diluted at 1:200 for two hours at room temperature.

#### Immunofluorescence

Tissue sections were fixed in PBS with 4% paraformaldehyde, permeabilized with 0.5% Triton X-100, saturated in 5% normal goat serum in PBS-T and hybridized with anti-mSDPLA_2 _polyclonal antibodies diluted at 1:100 in Dako Diluent (S3022) overnight at 4°C. After two washes, cells were hybridized for 2 h with a chromeo™-488-conjugated secondary antibody from rabbit (Abcam, Cambridge, UK) diluted at 1:200 in PBS. The fluorescence analysis were performed with BX41 Olympus upright microscope and pictures were taken with an Olympus C-5060 digital camera. Control experiments were carried out in absence of anti-mSDPLA_2 _antibody.

#### Electron microscopic study

Fragments of tissues, with a size of about 1 mm^3 ^were fixed in 2.5% glutaraldehyde for 2 hours, rinsed in sorensen's phosphate buffer and post-fixed for 1 hour 30 with 2% OsO_4_. Tissues were dehydrated in graded concentrations of alcohol, and then in propylene oxide. Thereafter, tissues were embedded in epoxy resin for 72 h at 60°C. Finally, ultrathin sections were cut and observed with a Jeol, Jem-1010 Electron Microscope.

## Conclusion

In this study we found two different localizations for production and secretion of the hepatopancreatic digestive PLA_2 _in two species of marine snails *Littorina littorea *and *Buccinum undatum*. While the first produced mSDPLA_2 _in digested cells, the second produced it in basophilic cells. Only these cells contained secretory materials exhibiting PLA_2_-like immunoreactivity. The hepatopancreas is adapted to increase gut surface area and it is a suitable compartment for lipid absorption.

## Abbreviations

mSDPLA_2_: marine snail digestive phospholipase A_2_; SDS-PAGE: sodium dodecyl sulfate-polyacrylamide gel electrophoresis; BSA: bovine serum albumin; pAbs: polyclonal antibodies; PBS: phosphate buffer saline; DAPI: 4',6-diamidino-2-phenylindole; OCT: Optimal Cutting temperature.

## Competing interests

The authors declare that they have no competing interests.

## Authors' contributions

ZZ carried out all the studies, analyzed the data and drafted the manuscript. NB helped with the analysis, discussion of the data and correction of the manuscript. PM and EG helped with the electron microscopic analysis and discussion of the data. SB helped with the correction of the manuscript. TR helped with the discussion of the data. HM and YG participated in the study design and helped to draft the manuscript. All authors have read and approved the final manuscript.
